# Effects of robot-assisted therapy on upper limb and cognitive function in patients with stroke: study protocol of a randomized controlled study

**DOI:** 10.1186/s13063-022-06361-2

**Published:** 2022-06-28

**Authors:** Yana Wang, Mingzhu Ye, Yujie Tong, Li Xiong, Xuejiao Wu, Chao Geng, Wen Zhang, Ziqi Dai, Wei Tian, Jifeng Rong

**Affiliations:** 1 The Center of Rehabilitation Therapy, the First Rehabilitation Hospital of Shanghai, No.349 Hangzhou Rd., Yangpu District, Shanghai, 200090 China; 2 Key Laboratory of Exercise and Health Science of Ministry of Education, Shanghai University of Sport, Shanghai, China; 3 The Neurorehabilitation Center, the First Rehabilitation Hospital of Shanghai, Shanghai, China

**Keywords:** Robot-assisted therapy, Upper limb function, Cognition, Stroke

## Abstract

**Background:**

Impairments in upper limb motor function and cognitive ability are major health problems experienced by stroke patients, necessitating the development of novel and effective treatment options in stroke care. The aim of this study is to examine the effects of robot-assisted therapy on improving upper limb and cognitive functions in stroke patients.

**Methods:**

This will be a single-blinded, 2-arm, parallel design, randomized controlled trial which will include a sample size of 86 acute and subacute stroke patients to be recruited from a single clinical hospital in Shanghai, China. Upon qualifying the study eligibility, participants will be randomly assigned to receive either robot-assisted therapy or conventional therapy with both interventions being conducted over a 6-week period in a clinical rehabilitation setting. In addition to comprehensive rehabilitation, the robot-assisted therapy group will receive a 30-min Armguider robot-assisted therapy intervention 5 days a week. Primary efficacy outcomes will include Fugl-Meyer Assessment for Upper Extremity (FMA-UE) and Mini-Mental Status Examination (MMSE). Other secondary outcomes will include Trail Making Test (TMT), Auditory Verbal Learning Test (AVLT), Digit Symbol Substitution Test (DSST), and Rey–Osterrieth Complex Figure Test (ROCFT). All trial outcomes will be assessed at baseline and at 6-week follow-up. Intention-to-treat analyses will be performed to examine changes from baseline in the outcomes. Adverse events will be monitored throughout the trial period.

**Discussion:**

This will be the first randomized controlled trial aimed at examining the effects of robot-assisted therapy on upper limb and cognitive functions in acute and subacute stroke patients. Findings from the study will contribute to our understanding of using a novel robotic rehabilitation approach to stroke care and rehabilitation.

**Trial registration:**

Chinese Clinical Trial Registry ChiCTR2100050856. Registered on 5 September 2021.

## Background

Stroke is the second leading cause of death and the third leading cause of disability globally [1, 2]. In China, there are approximately 3 million new stroke cases diagnosed annually which impose an enormous burden on family, society, and healthcare systems [3–5]. Motor and cognitive dysfunction are common complications among stroke patients following stroke and they often coexist [6]. Approximately 65% of post-stroke patients live with upper limb dysfunction [3] and up to 75% exhibit cognitive impairment [7–9]. The prevalence of concurrent impairment ranges from 10 to 23% [10]. Previous studies have shown that motor performance is associated with global cognition, memory, and executive function and that cognitive dysfunction may attenuate post-stroke mobility recovery [10–12]. Upper limb and cognitive dysfunction have also been shown to significantly affect the quality of life, functional recovery process, and social participation among patients after stroke [7, 13–15]. Therefore, efforts to identify effective post-stroke rehabilitation treatments aimed at improving upper limb and cognitive functions are of high clinical importance for stroke care.

There is currently a lack of high-quality evidence or clinical practice guidelines supporting the use of any specific intervention as a part of routine practice. Conventional rehabilitation approaches (e.g., manual therapy techniques, task-specific training, mental practice, and sensory intervention) [16] require patients to perform partial or full movements with both assistance and supervision of a therapist. Such interventions have been shown to be both time-consuming and labor-intensive in clinical rehabilitation settings [17].

Robot-assisted therapy is a novel approach for the provision of a safe, repetitive, intensive, and quantitative rehabilitation intervention [18]. Since its initial deployment in clinical research [19], there has been an increasing number of studies that have reported its potential in facilitating recovery of upper limb and cognitive functions [20–23]. A recent review concludes that robot-assisted training can significantly improve upper limb motor impairment among stroke patients [20]. However, other studies have indicated that robot-assisted training may not be clinically superior to usual care in helping improve functional outcomes after stroke [21, 22]. In addition, previous research has mainly focused on assessing the effects of robot-assisted training on motor performance and recovery, its effectiveness on improving cognitive function has not been fully investigated. A previous pilot study reported improved cognitive outcomes after robot-assisted training in patients with stroke. However, the lack of a control group has made it impossible to draw meaningful clinical conclusions from the study [23]. Therefore, in the current study, we aim to conduct a comparative study examining the efficacy of a robot-assisted therapy, compared with a conventional treatment therapy in improving upper limb and cognitive functions among acute and subacute stroke patients.

## Methods

### Objectives

The objectives of the study are:

1. To evaluate the effects of robot-assisted therapy on improving upper limb function in acute and subacute stroke patients
2. To test the effectiveness of robot-assisted therapy on improving cognition in acute and subacute stroke patients

### Trial design

**Fig. 1:**
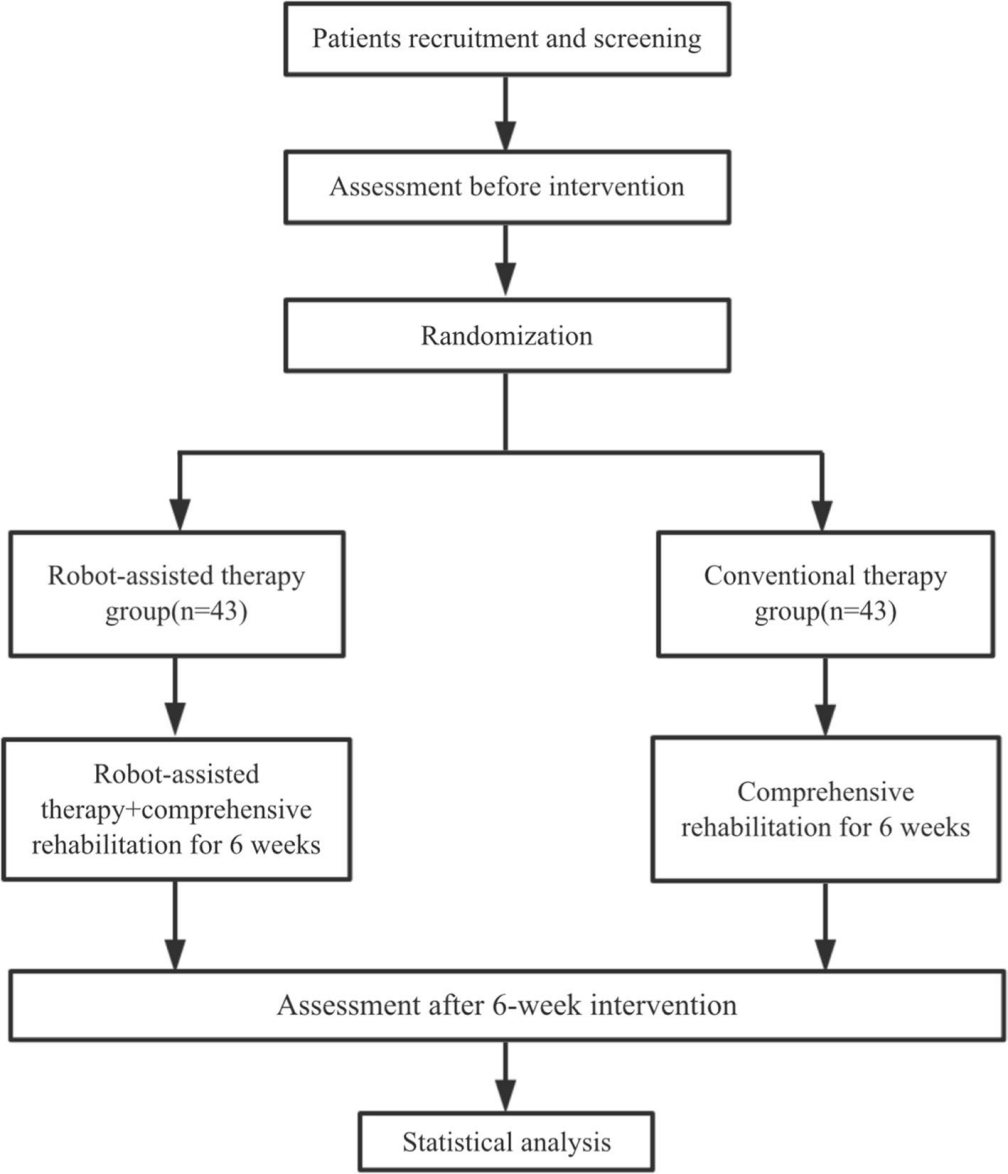
SPIRIT flow chart RCT study of patients with stroke

This study will involve a single-blinded, 2-arm, parallel design, randomized controlled trial with a 1:1 allocation ratio. Eligible patients will be randomly assigned to a robot-assisted therapy group or a conventional therapy group and receive their respective intervention treatment for 6 weeks. The study protocol is presented following the SPIRIT reporting guidelines [24], which is shown in Fig. 1.

### Recruitment and participants

Study participants will be recruited from the First Rehabilitation Hospital of Shanghai, China. Recruitment strategies will include posting research advertisements to the patients or their caregivers at multiples site in the hospital. Other recruitment methods include the use of word of mouth and social media. Interested patients or caregivers will be encouraged to contact research staff via telephone.

Patients will be included if they (1) are aged 18–65 years; (2) diagnosed with stroke by computed tomography or magnetic resonance imaging within 6 months following stroke onset [25]; (3) have a unilateral stroke for the first time; (4) have cognitive impairment (as determined via a Montreal Cognitive Assessment [MoCA] cut-off score of 26) [26, 27]; and (5) have severe upper limb functional deficits (Fugl-Meyer Assessment for Upper Extremity [FMA-UE]. 0–28) [23].

The exclusion criteria comprise the following: (1) severe visual, auditory, or speech impairments; (2) a history of epilepsy or serious heart, lung, liver, or kidney disease; (3) participation in robot-assisted training or other intervention programs in the past 3 months; and (4) inability to complete the entire intervention as required.

Participants will be considered withdrawal from the study if they (1) make a request to withdraw from the study; (2) show any serious adverse reactions to the robot-assisted therapy treatment; or (3) develop a serious disease that preclude them from continuing treatment.

### Procedure

**Fig. 2:**
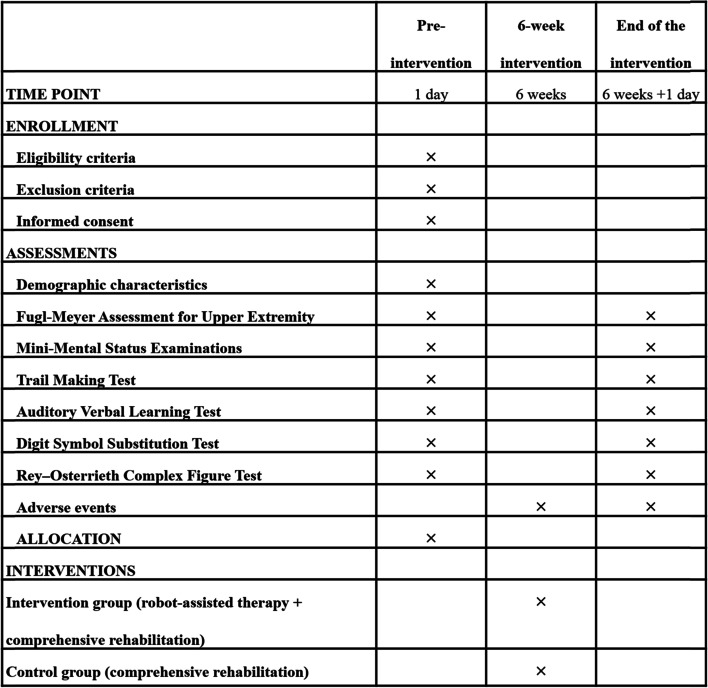
Schedule of enrollment, assessments, and interventions. The × symbol indicates the specific time point at which the corresponding measurement will be taken

The trial will be conducted in an academic setting located in a research laboratory. At an initial visit, patients and their caregivers will be provided with an introduction of the study protocol, which will include the detail about study procedures, intervention conditions, group randomization, and outcome assessments. All patients will provide written informed consent prior to participation in the study. Participants’ demographic and clinical characteristics, as well as the study outcomes, will be assessed at baseline. All outcome measures ascertained at baseline will be repeated at 6-week follow-up. The study protocol has been approved by the ethics committee of the First Rehabilitation Hospital of Shanghai. The schedule of enrollment, assessments, and interventions is shown in Fig. 2.

### Randomization and blinding

#### Sequence generation

Upon completion of baseline measurements, qualified patients will then be randomly assigned to one of the intervention groups. A computer-generated randomization sequence, generated by a statistician, will be used.

#### Allocation concealment mechanism

To ensure concealment, the sequence will be enclosed in sequentially numbered, opaque sealed envelopes. Research staff (LX) will strictly follow the pre-specified trial inclusion and exclusion criteria to determine whether the patients are eligible to participate in the study. The investigator (YT) will number the cases in order.

#### Implementation

On the day of the group assignment, the allocation schedule will be released to the project leader (JR) who will assign subjects to the study groups.

#### Blinding

To ensure trial blinding, outcome assessors (XW, CG) will be masked to group allocation and will not be involved in the administration of interventions. Due to the nature of our active interventions, participants and therapists will not be masked to group allocation.

#### Interventions

**Fig. 3:**
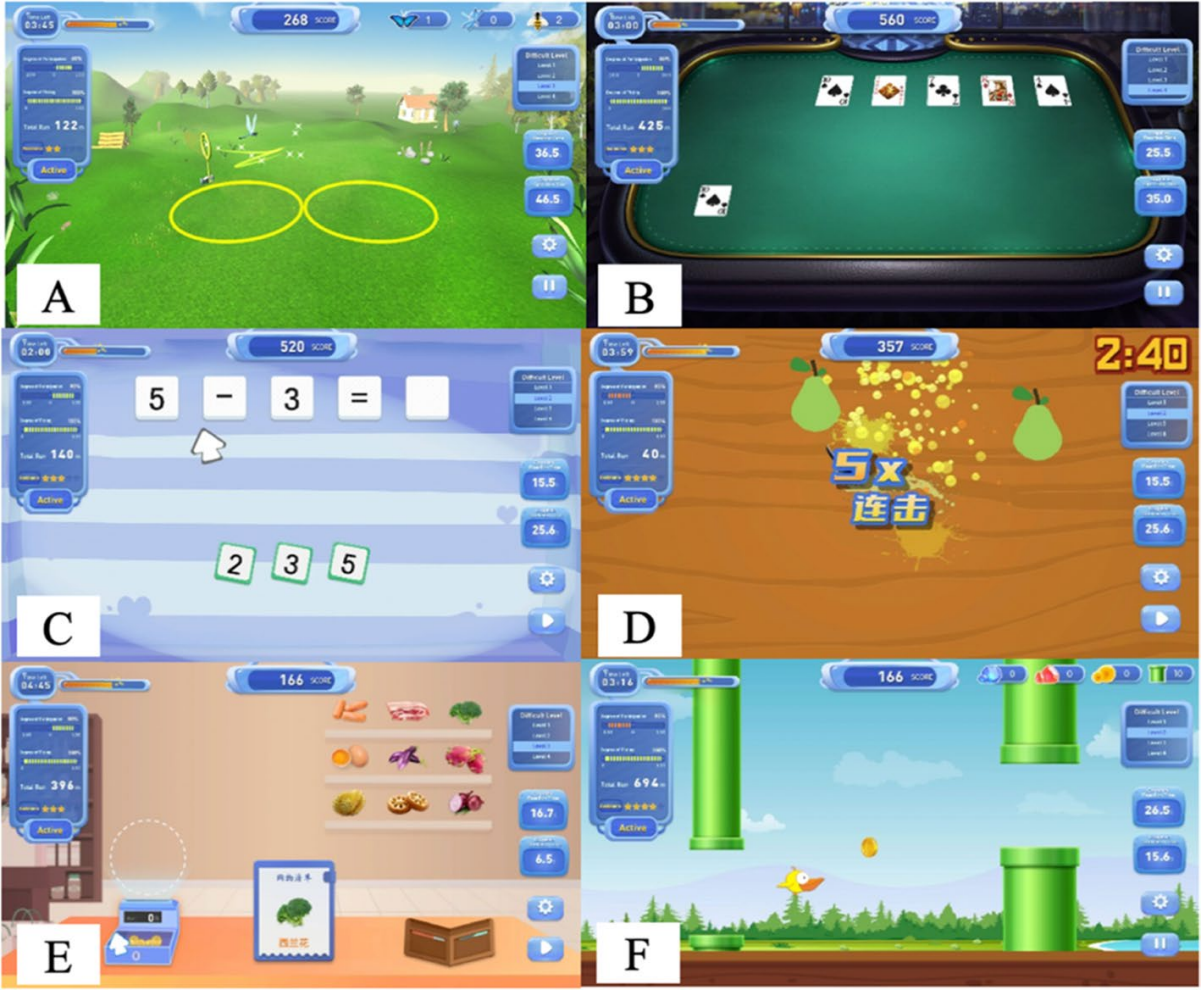
During treatment, patients will be asked to perform both motor and cognitive tasks shown in a game scene that generates real-time auditory and visual feedback. **A** Butterfly Capturing. **B** Playing Cards. **C** Calculation. **D** Fruit Ninja. **E** Supermarket Shopping. **F** Little Bird Flying

All patients will receive comprehensive rehabilitation therapy (45 min a day, 5 days a week over 6 weeks) that includes one-to-one physiotherapy and occupational therapy delivered by a certified physical and occupational therapist, respectively. In addition to the comprehensive therapy, the robot-assisted therapy group will receive physical training using the Armguider upper-limb robotic (ZD MEDTECH, Shanghai, China), which is an intelligent rehabilitation training system specially designed for people with upper limb or cognitive dysfunction. The system can provide passive, assistive, and active planar movements around the shoulder, elbow, and wrist joints. During treatment, participants will be asked to perform both motor and cognitive tasks shown in a game scene that generates real-time auditory and visual feedback. The planned games to be implemented in the study are described below (see also Fig. 3).

- Butterfly Capturing: Patients will be asked to control the net to capture a flying butterfly moving in different directions (this aims to assess visual-spatial ability and executive function)
- Playing Cards: A series of cards will be shown and then turned over. The patients will be asked to point out the cards with the same pattern (this aims to assess attention, scanning, memory, and delayed recall)
- Calculation: Patients will be asked to calculate the equations and move the correct answer to the designated position (this aims to assess computing ability)
- Fruit Ninja: Patients will be asked to observe the fruits that appear on the screen and cut them into sections while avoiding hitting obstacles (this aims to assess attention, graphic processing ability, response ability, and processing speed)
- Supermarket Shopping: Patients will be asked to buy the items on the list and pay the correct value of currency (this aims to assess visual scanning, selective attention, divided attention)
- Little Bird Flying: Patients will be asked to control a bird passing through pillars of different heights and avoid hitting obstacles (this aims to assess visual scanning and judgment ability)

Participants will spend 5 min on playing each of the six games described previously. When a game is completed, the system automatically and randomly switches to another game. The difficulty level of each game will be adjusted individually based on the patient’s ability and improvement. The intervention with the Armguider robot-assisted therapy will last 6 weeks, implemented at 30-min a day for 5 days a week. Patients will be monitored by research staff during the intervention period for potential adverse events including physical and/or emotional responses to games.

#### Primary outcomes

##### Fugl-Meyer Assessment for Upper Extremity

The FMA-UE assesses upper limb impairment and consists of 33 items. Performance is scored on a three-point ordinal scale (0, 1, or 2), with a maximum score of 66 points. The items are summed to provide a final score; a higher score indicates minimal or no impairment [28, 29].

##### Mini-Mental Status Examination (MMSE)

Global cognitive function will be assessed using the MMSE scale. MMSE is a single-page, 30-point test, which requires approximately 10 min to complete. It assesses different cognitive domains, including visuospatial/executive, naming, memory, attention, language, abstraction, delayed recall, and orientation; a higher score indicates better global cognitive function [30].

#### Secondary outcomes

##### Trail Making Test

Executive abilities will be evaluated through the TMT. The TMT includes parts A and B. In part A, patients are given a sheet of paper with circled numbers (from 1 to 25) and asked to draw lines to connect the numbers in ascending order, as quickly and accurately as possible. In part B, patients are again asked to connect the circles in ascending order, but are also instructed to alternate between numbers and letters. The time in seconds to complete each task is then analyzed [31, 32].

##### Auditory Verbal Learning Test

Memory will be measured using the AVLT. The assessor reads 12 two-character words randomly, and then the patients are required to recall the words. The test scores are calculated as follows: (1) AVLT Immediate Recall (AVLT-IR): the total of the correct recall words in the first three trials; (2) AVLT Short-term Delayed Recall (AVLT-SR): the sum of correct responses of free recall after short-term delayed (approximately 5 min); (3) AVLT Long-term Delayed Recall (AVLT-LR): the sum of correct responses of free recall after long-term delayed (approximately 20 min); (4) AVLT Category-cued Recall (AVLT-CR): the total of the correct answers under the category-cued recall condition; and (5) AVLT Recognition Test (AVLT-REC): the total of the correct answers in the recognition test [33].

##### Digit Symbol Substitution Test

Attention and processing speed will be assessed with the DSST. In this test, patients are given a sheet of paper with nine symbols and corresponding numbers from 1 to 9. Patients are instructed to write down each symbol under its corresponding number within 90 s; the number of correct answers are then scored [34].

##### Rey–Osterrieth Complex Figure Test

Visuospatial abilities and visual memory will be evaluated using the ROCFT. In this assessment, patients are asked to complete a complicated geometrical figure, which is comprised of 18 elements. Each element is scored on a two-point scale (0, 1, or 2) based on accuracy and placement, with a higher score indicating a better performance [35].

#### Other measures

These will include baseline demographic measures that assesse patient demographic characteristics (e.g., age, sex, stroke type [hemorrhagic/ischemic], educational level, affected side, time after stroke, handedness). In addition, history of disease will also be collected with a self-reported questionnaire.

### Statistical methods

#### Sample size and power

The sample size in this study is based on the expected difference between the two intervention groups in the outcome measure of FMA-UE score. We based our intervention effect from the existing literature on the effects of robotic training involving patients with stroke. With a predicted medium size effect, our power calculation indicated that, with a sample size of 78 stroke patients, we will have statistical power of 80% (at a two-tailed significance level of 0.05) to detect a between-group mean difference at an effect size of 0.65 for FMA-UE [36]. Power calculations were performed using G_*_Power free software (Version 3.1.9.4). Assuming a 10% attrition rate, we plan to enroll a total of 86 patients.

### Statistical methods for primary and secondary outcomes

With intention-to-treat, participants will be analyzed according to their assigned randomization group regardless of their participation status. Descriptive statistics will be performed to describe study population and patients’ demographical and clinical characteristics at baseline. Continuous variables and categorical variables will be presented as mean ± standard deviation or percentages, respectively. Baseline demographic descriptors will be compared across groups, using analysis of variance for continuous variables and the chi-square (or Fisher’s Exact) test for categorical variables.

For primary and secondary outcome analyses, we will use repeated measures analysis of variance (ANOVA) to compare pre- and post-intervention changes at 6-week follow-up in the repeated outcome measures (a within subject factor) between the two intervention groups (a between group factor). Analyses will be conducted with and without important adjustments for baseline covariates (e.g., age, gender, stroke type, affected side) using either ANOVA or analysis of covariance (ANCOVA) statistical models. Pre- and post-intervention change scores on the primary and secondary outcome variables and their 95 percent confidence intervals will be computed to determine the intervention effects. Two-sided *P* values of less than 0.05 will be considered to indicate statistical significance. No subgroup or supplemental analyses are planned. All statistical analyses will be performed using SPSS version 25 (IBM Corp., Armonk, NY, USA), with the significance level set at *p* ≤ 0.05.

#### Adverse event reporting and harms

Current literature on the use of robotic-assisted rehabilitation therapies has not reported any serious or major harms resulting from their administration to patients with stroke. However, we will take every precaution to monitor and record any adverse events (including minor, moderate, or serious events), either intervention or non-intervention related, during the entire course of our study and report them, in a timely manner, to the ethics committee and relevant regulatory agencies.

## Discussion

Current consensus from stroke survivors, caregivers, and health professionals has identified that upper limb and cognitive dysfunction in patients with stroke are among the top 10 priorities that need to be addressed in clinical research and practice [37]. Robot-assisted therapy is an emerging therapeutic approach for enhancing the recovery process and providing safe, repetitive, high-intensity, and task-specific rehabilitation interventions [38]. Previous studies have primarily focused on examining the effects of robot-assisted therapy on motor recovery function with relative little attention paid to the importance of cognitive function for stroke patients [18–23]. Cognitive function has been implicated as playing a crucial role in post-stroke mobility recovery and, therefore, needs to be taken into account as part of integral clinical treatment [10–12]. This trial is designed to specifically address this major gap in research and, more importantly, responds to current treatment needs that have been identified in clinical practice [37]. To understand potential cognitive benefits of robot-assisted therapy, we will use multiple assessment tools to comprehensively evaluate the cognitive domains ranging from global cognitive function to domain-specific cognition, including the areas of executive abilities, working and visual memory, attention, information processing speed, visuospatial abilities.

### Study innovation

The study is innovative in two notable areas. First, this is the very first study that specifically compares a novel robotic-assisted therapy against a conventional treatment therapy. Therefore, our approach represents a paradigm shift in current treatment options and, if successful, we will gain additional knowledge regarding the therapeutic value of a robotic-based rehabilitation approach to improving physical and cognitive function among post-stroke patients. Second, this is also the very first study that will simultaneously examine improvements in physical and cognitive functions, outcomes that have been identified as part of research priorities in supporting post-stroke recovery and improving quality of life in patients with stroke.

### Limitations

The study has limitations. First, the intervention time is considered brief due to the practical limits on the length of hospital stay by patients; Second, our study focuses on treating acute and subacute stroke patients with severe upper limb functional deficits. Therefore, the results from the trial may not be generalized to stroke patients with other functional impairments. Similarly, patients will be recruited from a single clinical hospital site which will also limit generalization.

## Conclusions

The present study addresses the limitations in current treatment options for stroke patients as well as aims to identify effective interventions that support optimal recovery and quality of life for the patients. We plan to conduct a randomized controlled trial to examine the effectiveness of robot-assisted therapy, relative to a standard care therapy, on improving upper limb function and cognition among acute and subacute stroke patients. Findings from this study are expected to contribute to our understanding of using a novel robotic rehabilitation approach to support post-stroke functional recovery among stroke patients with impairment in mobility.

## Trial status

The study registration number is ChiCTR2100050856 and currently in its recruitment phase.

## Abbreviations

FMA-UE: The Fugl-Meyer Assessment for Upper Extremity
MMSE: Mini-Mental Status Examination
TMT: Trail Making Test
AVLT: Auditory Verbal Learning Test
AVLT-IR: AVLT Immediate Recall
AVLT-SR: AVLT Short-term Delayed Recall
AVLT-LR: AVLT Long-term Delayed Recall
AVLT-CR: AVLT Category-cued Recall
AVLT-REC: AVLT Recognition Test
DSST: Digit Symbol Substitution Test
ROCFT: Rey–Osterrieth Complex Figure Test
MoCA: Montreal Cognitive Assessment
